# A new model order reduction strategy adapted to nonlinear problems in earthquake engineering

**DOI:** 10.1002/eqe.2802

**Published:** 2016-09-02

**Authors:** Franz Bamer, Abbas Kazemi Amiri, Christian Bucher

**Affiliations:** ^1^ Institute of General MechanicsRWTH Aachen University52062 AachenTemplergraben 64Germany; ^2^ Vienna Doctoral Programme on Water Resource SystemsVienna University of Technology1040 ViennaKarlsplatz 13/222Austria; ^3^ Center for Mechanics and Structural DynamicsVienna University of Technology1040 ViennaKarlsplatz 13/206Austria

**Keywords:** model order reduction, proper orthogonal decomposition, friction pendulum system

## Abstract

Earthquake dynamic response analysis of large complex structures, especially in the presence of nonlinearities, usually turns out to be computationally expensive. In this paper, the methodical developments of a new model order reduction strategy (MOR) based on the proper orthogonal decomposition (POD) method as well as its practical applicability to a realistic building structure are presented. The seismic performance of the building structure, a medical complex, is to be improved by means of base isolation realized by frictional pendulum bearings. According to the new introduced MOR strategy, a set of deterministic POD modes (transformation matrix) is assembled, which is derived based on the information of parts of the response history, so‐called snapshots, of the structure under a representative earthquake excitation. Subsequently, this transformation matrix is utilized to create reduced‐order models of the structure subjected to different earthquake excitations. These sets of nonlinear low‐order representations are now solved in a fractional amount of time in comparison with the computations of the full (non‐reduced) systems. The results demonstrate accurate approximations of the physical (full) responses by means of this new MOR strategy if the probable behavior of the structure has already been captured in the POD snapshots. Copyright © 2016 The Authors. Earthquake Engineering & Structural Dynamics Published by John Wiley & Sons Ltd.

## 1 Introduction

The evaluation of the response history of a structure in the time domain is one of the main topics in earthquake engineering and structural dynamics. It is a common practice to create simple structural models, for example, multistory shear frames, which should be able to describe the structural behavior and peculiarities of the real structure. This approach leads to useful results for the investigation of rather simple and uniform structures in order to come to meaningful engineering decisions regarding structural resistance. On the contrary, the analysis of complicated systems can require the application of nonlinear high‐order systems, as a characterization by a low dimensional structural model could lead to an oversimplification, that is, important motion patterns could be ignored. Therefore, an effective strategy is to obtain a set of a low number of ‘important’ equations of motion that approximates the high‐dimensional nonlinear dynamical system as accurately as possible, that is, model order reduction (MOR).

The solution of the nonlinear set of equations of motion in the time domain is realized by numerical algorithms, which require computational effort if the number of degrees of freedom (DOF) is high. Even the response calculation of linear systems can be expensive, as a factorization of the stiffness matrix is necessary to solve the eigenvalue problem and calculate the natural modes of vibration.

An alternative is to replace a high‐dimensional nonlinear set of equations of motion with a reduced set, providing the main dynamic behavior of the system to reach the required level of accuracy. MOR methods are used in many fields of research, where high‐dimensional systems are dealt with. Some review papers of MOR, especially for structural dynamic applications, are presented by Rega and Troga 1 and Koutsovasilis and Beitelschmidt 2. The classical but also effective method of modal truncation is well known in the field of earthquake engineering, which is however mainly applicable to linear systems.

This paper concentrates on a new MOR strategy based on the proper orthogonal decomposition (POD) method. The POD provides a low dimensional uncorrelated description (basis vectors), by which a high‐dimensional correlated process, for example, structural response, is spanned. Firstly, it was used as a statistical formulation in the papers of Kosambi 3, Karhunen 4 and Loeve 5.

The first paper regarding the field of structural dynamics was written by Cusumano *et al.* 6 in the early 1990s, who presented an experimental study of dimensionality in an elastic impact oscillator. In the papers of Feeny and Kappagantu (7, 8), a relation of the proper orthogonal modes to normal modes of vibration is investigated. Then they used the POD as they so call optimal modal reduction and exploit the benefits of the application of these modes in comparison with the linear natural modes. Furthermore, Kappagantu and Feeny (9, 10) investigated the dynamics of an experimental frictionally excited beam, and they verified that the proper orthogonal modes are efficient in capturing the dynamics of the system. Liang *et. al.* 11 discuss the realizations of the POD, that is, Karhunen–Loeve decomposition, principal component analysis, and singular value decomposition (SVD) and compare these three methods. Kerschen and Golivani 12 analyze the physical interpretation of the POD modes and its relation to the SVD, and they investigated POD based on auto‐associative neural networks 13.

The necessity to describe a high dimensional set by a small set of equations of motion, that is, MOR, has aroused interest mainly in the last two decades in the field of earthquake engineering (14, 15). Krysl *et al.* 16 deal with nonlinear MOR in finite element analysis. They introduce the POD for low‐order representations and point out the benefits concerning numeric integration, optimality, and robustness. Tubino *et al.* 17 investigated the seismic ground motion of the support points of a structure and classify the POD as a very efficient tool to simulate multivariate processes. Bucher 18 examined the stabilization of explicit time integration methods for analysis of nonlinear structural dynamics by modal reduction. Gutierrez and Zaldivar investigated in 19 how to handle the stability problem of explicit time integration by modal truncation methods more related to problems in earthquake engineering and structural dynamics, and they applied the Karhunen–Loeve decomposition, which is formally identical to the POD analysis, to capture the essential characteristics of nonlinear systems and provide experimental examples conducted on a shaker table 20. Bamer and Bucher 21 developed a MOR strategy applying the POD method for transient excited structures resting on one‐dimensional friction elements. This study presented a powerful combination of the POD and explicit time integration schemes.

The current work investigates the extension of the POD‐based MOR strategy, which is applicable to nonlinear systems in contrast to the method of modal truncation. The new strategy pursues the following objective: a low number of deterministic nonlinear modes (i.e., set of POD modes) is determined that defines a representative characterization of the structural behavior. Therefore, because of the information content of the full (or a part of the) time response of the structure to one representative excitation, a set of deterministic modes, that is, POD modes, is evaluated. Subsequently, this set of modes is utilized to project the equations of motion of a structure under different earthquake excitations onto POD coordinates, and thereafter, an order truncation is performed in a similar manner to the application of modal truncation to linear systems. The presentation of the novel MOR approach is accompanied by an explanatory and illustrative example, which supports basic understanding and visual insight into the method as well as an application to a realistic building structure.

It is essential to demonstrate this new strategy and its advantages by means of a practical application. Related to the first explanatory example, this application differs in complexity and the type of nonlinearities. The method is applied to the dynamic model of a realistic building structure. The building is erected on friction pendulum bearings for the sake of seismic isolation to minimize the transferred acceleration to the building during an earthquake. A three‐dimensional dynamic model of the base‐isolated structure is derived by implementing the FE model of the structure and the bi‐directional friction pendulum systems. The paper then deals with the nonlinear dynamic model of the base‐isolated structure. Thereafter, the new POD‐based MOR strategy and the example of its practical application is provided. Finally, the results and conclusions are outlined.

## 2 Earthquake Excitations

Within the presentation of the new strategy and the application to a realistic building structure, a set of six earthquake excitation records is chosen for the numerical demonstrations. The earthquake records are applied in fault‐parallel and fault‐normal directions. The excitation set includes the Bam earthquake (2003) in Iran and the following five representative events in California, USA: Northridge Rinaldi (1994), Imperial Valley (1979), Landers (1992), Loma Prieta (1989), and North Palm Springs (1986). Table 1 presents a list of the events taken from the Pacific Earthquake Engineering Research Center (PEER) 22. Fault‐parallel is defined in *x*‐direction and fault‐normal in *y*‐direction. Concerning the Bam event, only a one‐dimensional record is available; therefore, an excitation attack angle of 30° with respect to the *x*‐axis is chosen.

**Table 1:** Earthquake excitation list.

Event	Year	Location	*n*_*t*_	*T*	*d*	*M*	PGA
Bam	2003	Iran	1995	19.95	–	6.6	7.16
Imperial Valley	1979	California/Huston Road	3905	39.05	10	6.5	4.79
Landers	1992	California/Barstow	4932	49.32	36	7.3	4.13
Loma Prieta	1989	California/Gilroy	2507	25.07	12	7.0	9.51
North Palm Springs	1986	California/Palm Springs	6009	60.09	6.7	6.0	9.99
Northridge Rinaldi	1994	California/Newhall	1200	12.00	6.7	6.7	5.23

*n*_*t*_[ − ] number of time steps, *T*[*s*] duration of the record, *d*[*k* *m*] distance from epicenter, *M* moment magnitude, and PGA [*m*/*s* ^2^] peak ground acceleration.

## 3 Nonlinear Model Order Reduction and the POD‐Mathematical Formulation

The *n*‐dimensional set of equations of motion of a structure with nonlinear material behavior excited by horizontal components of ground acceleration is expressed as (cf. Chopra 23)

(1) $$M\ddot{x}+C\dot{x}+R(x)=-M\left(f_{x}{\ddot{x}}_{g}+f_{y}{ÿ}_{g}\right),\;$$

where **M** and **C** are mass and damping square matrices of order *n* and **R**(**x**) is the nonlinear internal restoring force vector dependent on the displacement **x** with the dimension *n* × 1. The right hand side of the set of equations of motion describes the earthquake excitation term, while ${\ddot{x}}_{g}$ and ${ÿ}_{g}$ denote the ground acceleration in *x* and *y* direction, and **f**_*j*_,(*j* = *x*,*y*) are the influence vectors in the corresponding direction, that is,

(2) $$f_{x}(x_{i})=1\;,\;f_{y}(y_{i})=1\;,\;i=1\ldots n,\;$$

at the global *x* and *y* DOF of all nodes, whereas the other components of *f*_*x*_ and *f*_*y*_ are zero. Thus, *i* describes the number of nodes of the FE discretized structure. This general approach indicates that in this paper, the ground acceleration in the corresponding direction, that is, *x* and *y* direction, is equal in all structural support points. In the following equations, the term on the right hand side of the set of equations of motion ((1)) is denominated by **F**(*t*), which has the unit of a force.

Nonlinear systems, as they are depicted in Equation ((1)), have generally to be solved by the application of a numerical algorithm, that is, a step by step procedure in the time domain in order to obtain the response of the structure as a function of time. The application of a numerical method inevitably leads to the existence of computational effort if *n* is a large number. Therefore, the approximation by a low‐dimensional description of the system seems to be useful, namely the application of MOR.

The main goal of MOR techniques is primarily to define a transformation matrix $T\in R^{n\times m},\;m\ll n$ to approximate the displacement vector $x\in R^{n}$ through a reduced coordinate vector $q_{r}\in R^{m}$ by the relation (cf. Koutsovasilis and Beitelschmidt 24)

(3) $$x=Tq_{r},\;$$

such that the dynamic properties of the system are preserved, and the error is small. The notation of the variable m∈N is the dimension of the reduced subspace.

The projection of the nonlinear system defined by Equation ((1)) onto that subspace leads to another second‐order ordinary differential equation (cf. Koutsovasilis and Beitelschmidt 24)

(4) $$m_{r}{\ddot{q}}_{r}+c_{r}{\dot{q}}_{r}+r=f_{r},\;$$

where *m*_*r*_ = *T* ^*T*^ **M** **T** and $c_{r}=T^{T}CT\in R^{m\times m}$ are mass and damping matrices, and $f_{r}=T^{T}F(t)\in R^{m\times 1}$ is the force vector in the reduced subspace. It should be noted that the reduced system matrices *m*_*r*_ and *c*_*r*_ are generally not diagonal. The vector of the restoring forces in the reduced subspace is

(5) $$r=T^{T}R(x)=T^{T}R(Tq_{r}).\;$$

Consequently, one necessity of nonlinear MOR is the evaluation of the vector of the restoring forces in the physical (full) coordinate at every time step.

Modal truncation is a widely‐used tool and an effective method for order reduction of linear systems in the field of earthquake engineering. An accurate approximation of the response history is achieved by applying a small number of lower structural modes proportional to the number of DOF. An early and successful application of modal truncation to problems involving small local nonlinearities is presented in 25 and 26. In this work, the objective is to find a new strategy that is applicable to nonlinear systems in a similar manner to modal truncation. The approach is to define a set of deterministic modes that can be evaluated from the information of an existing response history of the structure. Consequently, this set of modes contains nonlinear motion patterns if the structure shows nonlinear response behavior to the excitation.

The proposed strategy is established based on the theory of the POD method. Generally, the POD (cf. 11, 27, 28) is a straightforward approach to obtain a low‐dimensional uncorrelated process from a correlated high dimensional or even infinite‐dimensional process. Holmes *et al.* 28 examined the theoretical background of the POD and its properties profoundly. In the following sections, the mathematical basics of the POD are discussed shortly, but as the paper is more targeted to the strategic approach in earthquake engineering, the attention to the mathematical background and the numerical problems are limited to an essential minimum.

The aim of the POD is to find a set of ordered orthonormal basis vectors in a subspace so that samples in a sample space are expanded in terms of *l* basis vectors in an optimal form. This means that the POD is able to find an orthonormal basis, which describes an observation vector in a subspace better than any other orthonormal basis can do. A measure for this problem is the mean square error (cf. Qu 29)

(6) $$E\left\{∥x-x(l){∥}^{2}\right\}\leq E\left\{∥x-\hat{x}(l){∥}^{2}\right\},\;$$

where $x\in R^{n\times 1}$ is the random vector, **x**(*l*) is the approximation of this random vector in an *l*‐dimensional POD subspace, and $\hat{x}(l)$ is the approximation of the random vector by any other possible orthonormal basis. Therefore, the random vector can be expressed as (cf. Qu 29)

(7) $$x=\Phi_{p}q_{p}\;,\;\Phi_{p}=[\varphi_{p,1},\varphi_{p,2},\ldots ,\varphi_{p,s}]\;\text{and}\;q_{p}=[q_{p,1},q_{p,2},\ldots ,q_{p,s}],\;$$

where ***φ***_**p**,**i**_ are the POD modes, *q*_*p*,*i*_ denote the coordinates in the POD subspace, and *s* is the number of realizations of the random vector (also called snapshots). This leads to an optimization problem with the following objective function (cf. Qu 29):

(8) $$\varepsilon^{2}(l,t)=E\left\{∥x-x(l){∥}^{2}\right\}\to min$$

subject to the orthonormality condition (cf. Qu 29)

(9) $$\varphi_{p,i}^{T}\varphi_{p,j}=\delta_{ij}\;(i,j=1,2,\ldots ,s)\;.$$

The transformation into the *l*‐dimensional POD subspace is a truncation of the first *l* lower POD modes (cf. Qu 29)

(10) $$x(l)\approx \Phi_{p}q_{p}\;,\;\Phi_{p}=[\varphi_{p,1},\varphi_{p,2},\ldots ,\varphi_{p,l}]\;,\;l<s\ll n.\;$$

## 4 The New Approach

In this section, the reader's attention is drawn to the methodical approach. Therefore, a simple and illustrative two‐dimensional structural example subjected to a set of ground motions goes along with the presentation of the new MOR strategy. Earthquake excitation records are presented in Section 2, respectively in Table 1.

**Figure 1:**
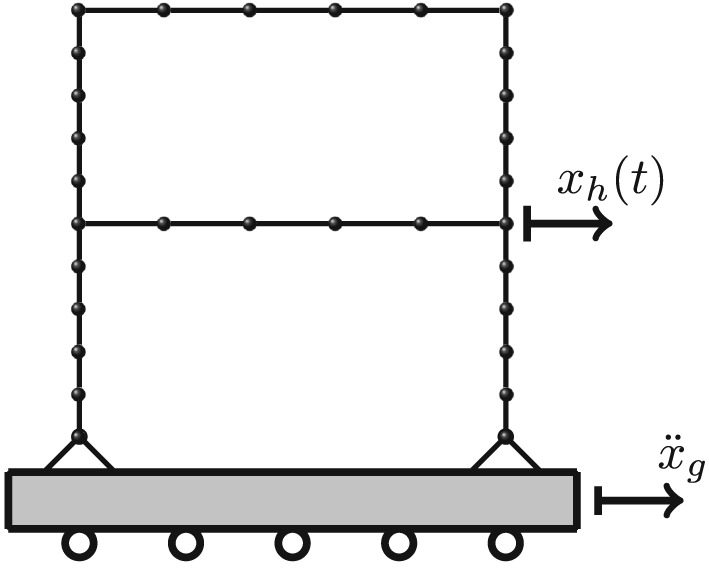
Test example: two‐story frame structure subjected to ground motion.

**Figure 2:**
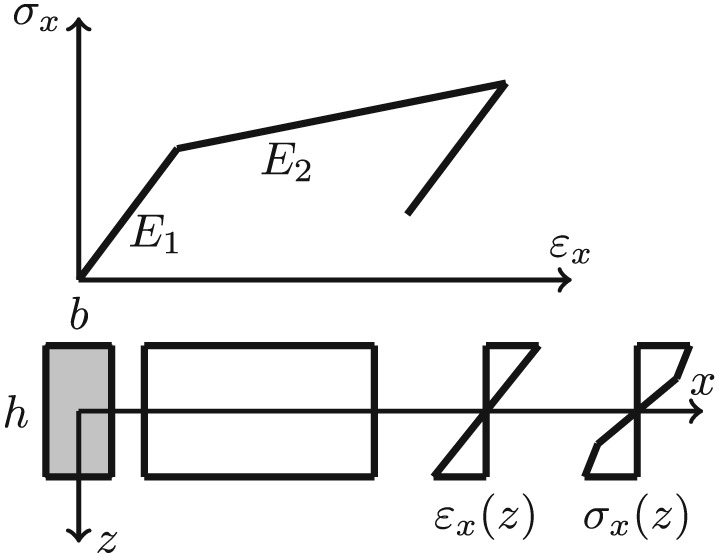
Elasto‐plastic stress strain relation of the beam in local element coordinates.

The academic example concerns a two‐story frame system as an academic example modeled by nonlinear elasto‐plastic beam elements. The geometrical discretization is shown in Figure 1. The frame span is *l* = 6[*m*], and the height of it is *h* = 4[*m*]; consequently, the total height is eight 8 m. The columns as well as the beams of the frame structure are discretized by five elements, which leads under consideration of the boundary conditions shown in Figure 1, to a total number of 86 DOFs. Elasto‐pasticity is realized by a bilinear stress‐strain curve with kinematic hardening in axial beam direction as shown in Figure 2. Fictitious material parameters are assumed in this academic example to induce large plastic deformations in order to illustrate a clear visualization of the nonlinear behavior and accordingly a good insight into the nonlinear MOR procedure. The paramters of the elasto‐plastic material are the following: Young's modulus $E=2.1\times 10^{11}\;\left[\frac{N}{m^{2}}\right]$, Poisson's ratio 0.3[ − ], elastic yield limit $2.4\times 10^{7}\;\left[\frac{N}{m^{2}}\right]$, with 5% post‐yielding stiffness ratio. The cross section of the column is quadratic (0.1 × 0.1[*m* ^2^]), and the cross section of the beam is rectangular (0.1 × 0.3[*m* ^2^]).

In addition to the presented nonlinear system with hysteretic material behavior, the equivalent structure with linear material parameters is presented. The linearized material parameter is equal to the initial stiffness of the elasto‐plastic material model presented in Figure 2, that is, Young's modulus $E=2.1\times 10^{11}\;\left[\frac{N}{m^{2}}\right]$.

The extended approach of the new MOR strategy is based on the mathematical formulations of the POD in Equations ((6))–((10)). In structural dynamics, systems are discretized in space and time; therefore, the numerical realization of the proposed strategy is implemented based on the following straightforward algorithm.

**Figure 3:**
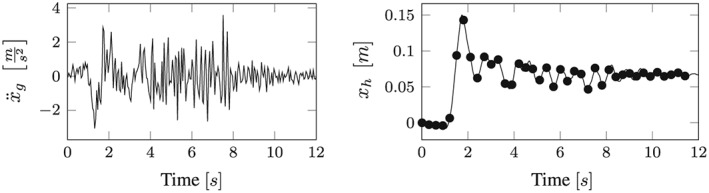
Left side: Bam earthquake excitation, excitation function in the snapshot time period; Right side: Snapshot response function *x*_*s*_(*t*) of the nonlinear system (solid line), snapshots (dots).

Firstly, an a priori response identification, that is, the realization of a random vector **x**(*t*), is evaluated, where *t* is in a limited time interval *t*_0_≤*t*≤*t*_1_. Therefore, the response to one excitation in the specific time period is computed, that is, Equation ((1)) is solved numerically in the defined time period. This analysis is performed in the physical coordinate **x**. Consequently, this constitutes dependent on the size of the time period *t*_1_ − *t*_0_(snapshot time period) and the number of DOFs of the system, the most time consuming part of the procedure. According to the actual example, for the linear as well as the nonlinear system, the Bam earthquake in Table 1 is chosen as the representative event. The snapshot time period is here limited by *t*_1_ = 0[*s*] and *t*_2_ = 12[*s*]. This means that the largest possible time window for the snapshot response identification is chosen; therefore, the maximum possible response information is captured here. Although in order to increase effectivity of the algorithm, a representative deterministic basis can be obtained by concentrating on the strong‐motion phase of the earthquake because the relevant nonlinearities will be activated during that phase, but then it is more likely that insufficient POD response bases for MOR transformation matrix for the upcoming analyses are acquired. The time history of the excitation is shown in the left subplot of Figure 3.

The snapshot response function *x*_*s*_(*t*), that is, the random vector, is realized by *s* observations (snapshots) at different time instances (cf. Han and Feeny 30) within the snapshot time period

(11) $$X_{s}=\left[x_{t_{1}},x_{t_{2}},\ldots ,x_{t_{s}}\right]=\left(\begin{array}{lll} x_{1}(t_{1}) & \cdot \cdot \cdot & x_{1}(t_{s})\\ \cdot \cdot \cdot & \cdot \cdot \cdot & \cdot \cdot \cdot \\ x_{n}(t_{1}) & \cdot \cdot \cdot & x_{n}(t_{s}) \end{array}\right).\;$$

It is of utmost importance to capture nonlinear deformation patterns in order to create the possibility to depict possible nonlinear reactions in forthcoming calculations. In the specific example, 40 observations (snapshots) in equidistant time instances are taken into account from the snapshot response function for the a priori identification of the overall response behavior. Those observations of the nonlinear system are specified by dots in the right subplot of Figure 3, to be distinguished from the overall response within the time period of analysis. According to the linear system, the computation of the snapshots is realized by the same procedure. As a result, it is also necessary here to capture the main motion patterns according to possible future response histories. However, it can be observed that for the system identification of the linear response, the required number of snapshots is considerably smaller than for the nonlinear system response.

If ***μ*** is the expectation of all observations, then the sample covariance matrix **Σ_*s*_** of this random vector, which is realized by the observation matrix, is defined by (cf. Kerschen *et al.* 31)

(12) $$\Sigma_{s}=E\{{\left(x-\mu \right)}^{T}(x-\mu )\}.\;$$

The POD modes and the POD values are defined by the eigensolution of the sample covariance matrix. If the data have zero mean, the covariance matrix is (cf. Kerschen *et al.* 31)

(13) $$\Sigma_{s}=\frac{1}{s}{X_{s}}^{T}X_{s},$$

and the POD is realized by the SVD of the observation matrix *X*_*s*_. The POD modes ***φ***_**p,i**_ are equal to the left singular vectors and the POD values *λ*_*p*,*i*_ to the singular values of *X*_*s*_, which are all real and positive and arranged in a rectangular diagonal matrix in descending order. The energy, which is contained by the snapshot matrix, is defined by the summation of the POD values, that is, $V=\sum_{i=0}^{s}\lambda_{p,i}$. As a consequence, the energy ratio of the *i*_*th*_ POD mode is (cf. Kerschen *et al.* 31)

(14) $$V_{i}=\frac{\lambda_{p,i}}{\sum_{i=0}^{s}\lambda_{p,i}}.\;$$

**Figure 4:**
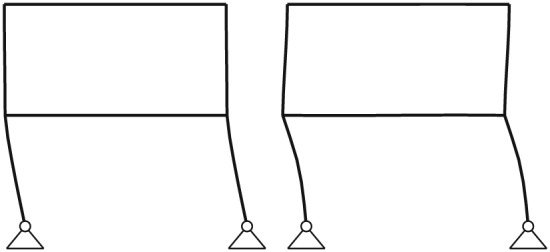
First two proper orthogonal decomposition modes.

**Figure 5:**
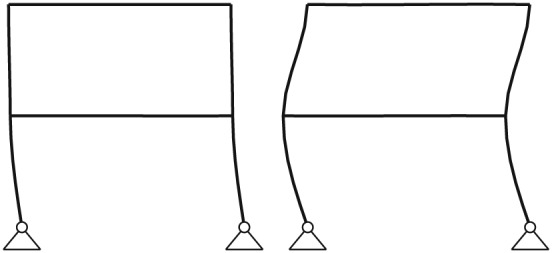
First two natural modes.

In structural dynamics applications, the sum of only a few POD values often captures the main part of the total energy included in the observation matrix, which reflects the big advantage of the POD, that is, the property of optimality with respect to energy in a least square sense. In the present demonstration, 99% of the total energy is captured by only two deterministic modes. The first two evaluated POD modes of the nonlinear system are depicted in Figure 4. In comparison, the natural modes of vibration, which create the basis for the classical modal truncation method are shown in Figure 5. They are computed according to the eigenvalue problem (**K** − *ω* ^2^ **M**)Φ = **0**, where **K** is the linearized initial stiffness matrix according to the aforementioned assumption.

As depicted in Figure 4, the POD modes can obviously represent nonlinear displacement patterns, which are expected to occur in this illustrative example, for example, plastic deformation areas around the joints of the frame system. But rather these deformation patterns are not representable by the first two linear natural modes of vibration depicted in Figure 5. Therefore, according to the nonlinear response, an accurate representation of the snapshot response function *x*_*s*_(*t*) is expected by the representation of only the two deterministic POD modes, but a significant error by the representation of the first two natural modes of vibration is expected.

Following, the transformation into the reduced subspace is performed in the same manner, as if the classical method of modal truncation would be applied to linear systems. The low‐order set of equations of motion is then

(15) $$\tilde{M}{\ddot{q}}_{P}+\tilde{C}{\dot{q}}_{P}+\tilde{R}=\tilde{F},\;$$

where $\tilde{M}=\Phi_{P}^{T}M\Phi_{P}$ and $\tilde{C}=\Phi_{P}^{T}C\Phi_{P}$ are mass and damping matrices, and $\tilde{F}=\Phi_{P}F$ is the excitation vector in the POD reduced subspace. The reduced vector of the inner restoring forces $\tilde{R}$ is still dependent on the displacement in the physical coordinate **x**,

(16) $$\tilde{R}=\Phi_{P}^{T}R(\Phi_{P}q_{P})=\Phi_{P}^{T}R(x).\;$$

**Figure 6:**
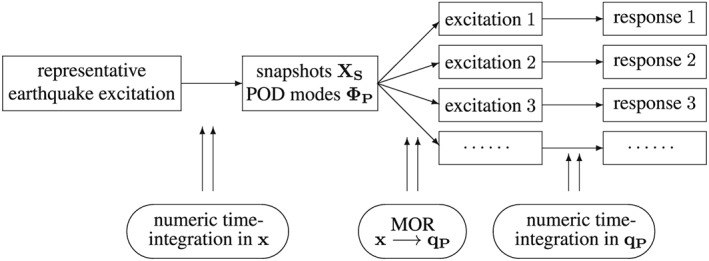
Approach of the new strategy.

Therefore, the vector of the inner restoring forces **R** **(** **x** **)** has to be evaluated from the physical model in the full‐order coordinates in every calculation time step. According to the linear POD reduced system, the vector of the inner restoring forces is described by the relation $\tilde{R}=\tilde{K}q_{P}$, where $\tilde{K}=\Phi_{P}^{T}K\Phi_{p}$ is the stiffness matrix in the POD reduced subspace. In this case, no updating in every time step of the inner restoring forces has to be performed. Furthermore, the equations of motion in the reduced‐order set are not decoupled and have to be solved numerically. Finally, after the time integration is finished, the solution vector, *q*_*P*_, dependent on time is transformed back into the physical coordinate **x**. Concerning the representative excitation, a comparison of the full benchmark solution (snapshot response function *x*_*s*_(*t*)) makes sense in order to verify the quality of the low‐dimensional representation of the POD modes. If the approximation of the benchmark solution (in this example, Bam earthquake response) is satisfactory, then the reduced solution to the whole set of the remaining earthquakes is calculated by application of these deterministic modes. If the approximation is not sufficient, one possibility is to increase the number of snapshots within the snapshot time period; another one is to change the start and end time instances *t*_0_ and *t*_1_ of the snapshot response function in order to improve the response information quality of the snapshot matrix (remark: in this paper *t*_0_ and *t*_1_ are already the beginning and end time step of the Bam excitation record). The visualization of the novel approach is depicted in Figure 6.

**Figure 7:**
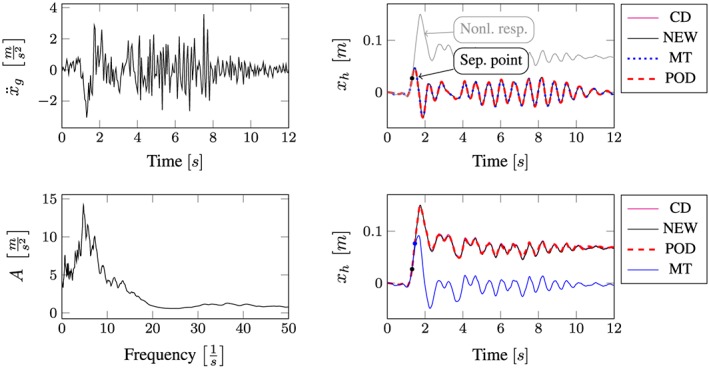
Results Bam earthquake. Top left: Bam earthquake time history; bottom left: peak acceleration response spectrum *ζ* = 0.04; top right: linear response functions; bottom right: nonlinear response functions. [Colour figure can be viewed at wileyonlinelibrary.com]

**Figure 8:**
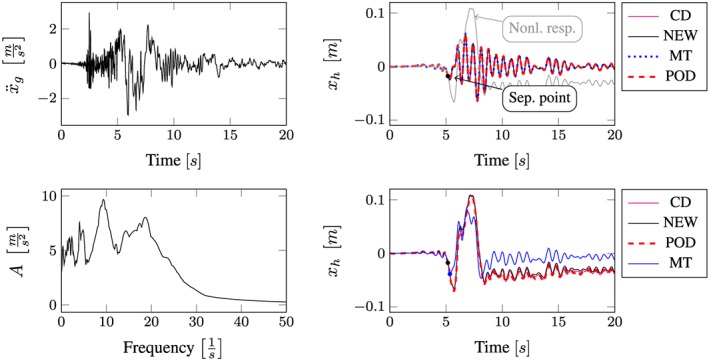
Results Imperial Valley earthquake. Top left: Imperial Valley earthquake time history; bottom left: peak acceleration response spectrum *ζ* = 0.04; top right: linear response functions; bottom right: nonlinear response functions. [Colour figure can be viewed at wileyonlinelibrary.com]

**Figure 9:**
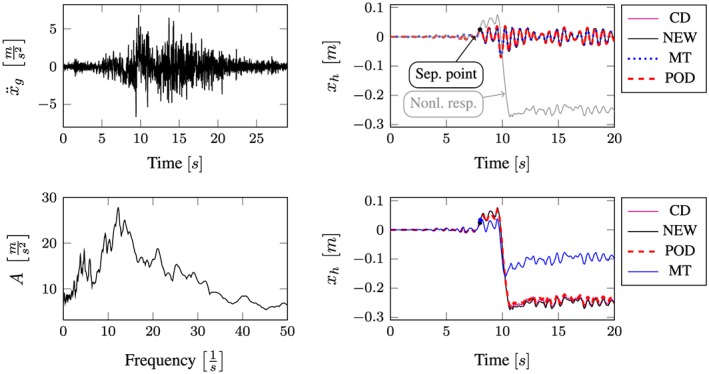
Results Landers earthquake. Top left: Landers earthquake time history; bottom left: peak acceleration response spectrum *ζ* = 0.04; top right: linear response functions; bottom right: nonlinear response functions. [Colour figure can be viewed at wileyonlinelibrary.com]

**Figure 10:**
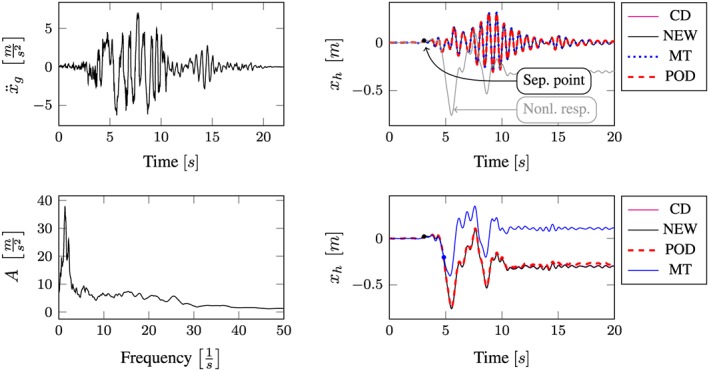
Results Loma Prieta earthquake. Top left: Loma Prieta earthquake time history; bottom left:peak accleration response spectrum *ζ* = 0.04; top right: linear response functions; bottom right: nonlinear response functions. [Colour figure can be viewed at wileyonlinelibrary.com]

**Figure 11:**
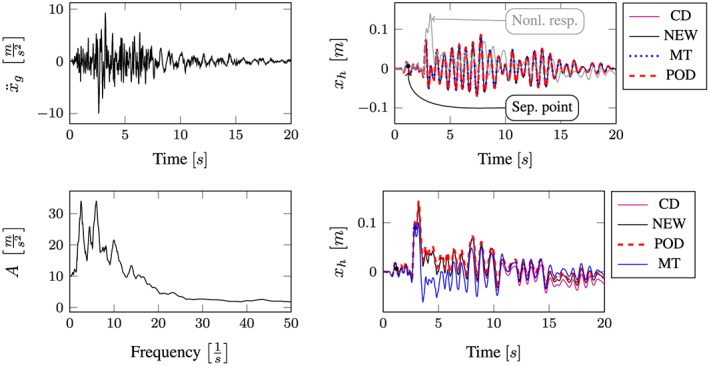
Results North Palm Springs earthquake. Top left: North Palm Springs earthquake time history; bottom left: peak acceleration response spectrum *ζ* = 0.04; top right: linear response functions; bottom right: nonlinear response functions. [Colour figure can be viewed at wileyonlinelibrary.com]

**Figure 12:**
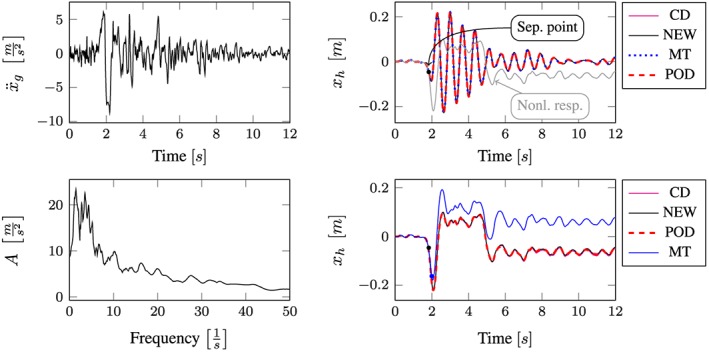
Results Northridge earthquake. Top left: Northridge earthquake time history; bottom left: peak acceleration response spectrum *ζ* = 0.04; top right: linear response functions; bottom right: nonlinear response functions. [Colour figure can be viewed at wileyonlinelibrary.com]

All computational results according to the whole excitation set presented in Table 2 are depicted in Figures 7, 8, 9, 10, 11, 12. Within each of the response computations of the linear, as well as the nonlinear system, two different integration algorithms were chosen for the integration procedure in the physical (full) coordinate (86 DOFs), that is, the implicit Newmark integration (NEW) and the explicit central difference scheme (CD). They serve as reference solutions to the suggested new POD strategy, which is compared with the classical modal truncation method (MT). All time response functions are compared according to the horizontal displacement in the right corner of the first floor of the frame structure, *x*_*h*_(*t*), depicted in Figure 1. For the reduced computations, 2 DOFs concerning the new POD strategy (99.9% of the total energy of the snapshots is captured) and 4 DOFs concerning the classical MT strategy (according to an assumed cut‐off frequency of 30 Hz dependent on a qualitative investigation of the fourier transforms of the events (mode 1: 1.46 [Hz], mode 2: 6.45 [Hz], mode 3: 20.38 [Hz], mode 4: 23.65 [Hz], mode 5: 40.15 [Hz]) are considered.

According to the proposed strategy in Figure 6, the Bam earthquake serves as the representative event (Figure 3). A test run of the reduced order model over the time period of the representative Bam earthquake shows that the deterministic POD modes can represent the nonlinear (elasto‐plastic) response time history. This is shown in the top right and the bottom right subplot in Figure 7. According to Figure 7, both the linear and the nonlinear POD response functions approximate the full reference solutions (NEW and CD) accurately. Therefore, the deterministic set of POD modes is able to represent nonlinear (plastic) deformation patterns. Consequently, the chosen number of 40 snapshots within the chosen snapshot time period (Figure 3) is apparently sufficient for this example. Figures 8, 9, 10, 11, 12 present the numerical evaluation of the rest of the earthquake set shown in Table 2, that is, the comparison of the reduced order models with the full benchmark solutions. In each of the linear and nonlinear response evaluations, it is shown that the POD responses provide reliable approximations of the benchmark solutions (response functions are overlapping with each other). This underlines the robustness of the new strategy according to changes in the excitation if the deterministic POD modes are evaluated based on a different excitation history (Figure 3).

According to the linear computations, both strategies show accurate results (see the top right subplots of Figures 7, 8, 9, 10, 11, 12). In this special linear elastic case, the method of modal truncation shows slight advantages, which is not surprising as no snapshot response function in the physical coordinate must be evaluated. However, a distinct advantage is that the POD strategy does not require an investigation of the Fourier transform of the excitation in order to determine the cut‐off frequency for estimating the truncation of the natural basis modes. On the contrary, the POD method fulfills the requirement in Equations ((6)) and ((7)), where an optimally truncated basis is defined. Therefore, dependent on the required level of accuracy, an automatic truncation based on the energy content per mode is performed (Equation ((14))). According to the nonlinear system (see the bottom left subplots of Figures 7, 8, 9, 10, 11, 12), the modal truncation responses show a severe deficit with respect to the exact solution, because the natural modes (as earlier presented within the Figures 4 and 5) cannot detect the nonlinear deformation patterns. On the contrary, the POD strategy, as already discussed previously, shows reliable approximations. Therefore, in the presence of strong nonlinearities, the new POD strategy has sizeable advantages over the classical method of modal truncation. However, within a limited range, the modal truncation method is reliable. In the top right subplots of Figures 7, 8, 9, 10, 11, 12, the separation point (sep. point, black mark) defines the time instant, when, according to Figure 2, the first nonlinear effect occurs (yielding of the material). Before this time instant, each response line (CD, NEW, POD, and MT) is the same. This is not surprising, because this is the simple linear‐elasic solution. Afterwards, as presented in the top right subplots of Figures 7, 8, 9, 10, 11, 12, the nonlinear yielding effect is shown by the drift‐off of the light gray nonlinear reference responses in these subplots (nonl. resp.), which is equivalent to the nonlinear Newmark response of the bottom right subplots of these figures. Here, the black mark also shows the separation point of the linear and the nonlinear response, and additionally, the qualitative separation point of the modal truncation solution (blue mark), that is, where the modal truncation solution drifts off. Therefore, it is clearly shown that there is a limited range of nonlinearity, where the modal truncation response also presents useful results – after the modal truncation separation point, a severe drift‐off of this solution function is observed.

The first substantial numerical benefit of the proposed strategy arises from the combination of the MOR and the application of an explicit time integration method, such as the second order central difference scheme. The time consuming part of the computation is the evaluation of the inner restoring force vector, which has to be evaluated in every time step on Gauss integration point level in the physical (full) coordinate according to Equation ((16)). This recalculation cannot be avoided. The application of an explicit time integrator to the full system leads inevitably to considerably small integration time steps, which have to be smaller than a certain value (critical time step for the central difference scheme $\Delta t_{cr}=\frac{2\pi }{\omega_{n}}$, where *ω*_*n*_ is the highest eigenfrequency). Therefore, a huge number of time steps has to be processed within one integration run and consequently, a high number of the expensive recalculations of the inner restoring force vector **R** **(** **x** **)**. For the integration in the POD reduced subspace the critical time step is considerably larger (according to this example in the paper even larger than the measurement time step of the earthquake data), which leads to a stabilization of the procedure and as a consequence, to approximately a 500‐fold increase of speed compared with the full central difference integration method (CD). These numerical issues are discussed in a similar manner by Gutierrez and Zaldivar 19 applied to modal truncation. For the numerical benefit of the combination of the basic POD method with explicit numeric time integration, the reader is referred to Bamer and Bucher 21. It would also be possible to implement an implicit integration scheme (i.e., the Newmark method with constant acceleration assumption) in the reduced subspace, which is under special circumstances convergent for all possible time steps. However, the application of this integration scheme requires the iterative Newton Raphson procedure, following, in each timestep of the reduced system, the restoring force vector *R* must be computed several times (modified Newton Raphson method). If the classical (not modified) Newton Raphson method is applied, the number of iterations is minimized, but then in every iteration procedure the tangential stiffness matrix in the full space is to be evaluated, which has then again transformed into a tangential stiffness matrix in the reduced subspace. Therefore, the explicit central difference scheme is chosen, which is stabelized by the model order reduction process. Generally, each integration scheme can be applied for the realization of the POD reduced strategy, and of course, the choice of the integration scheme is also dependent on the level of high dimensionality, type of nonlinearity, and so on.

The second big numerical advantage of the new strategy is that the actual time consuming process, which is the evaluation of the snapshot matrix, is only executed once at the beginning of the whole calculation procedure. This a priori assumption of nonlinear mode patterns makes sense if the excitations show physical ‘similarities’, which is the case in earthquake analysis, where a considerably small number of low frequency modes is mainly affected.

The practical application of this novel approach in earthquake engineering analyses can be realized in a straightforward manner. Earthquake design codes frequently specify the earthquake loading in terms of response spectra rather than actual acceleration records, but this is directly usable only in the case of linear system behavior. For the analysis of nonlinear systems, several response spectrum compatible artificial earthquake accelerations (as specified, e.g., in Eurocode 8) can be generated, see for example, Ref. 32. Based on these artificial accelerations, the proposed model order reduction approach is carried out exactly as described in this section.

### 4.1 Error evaluation

**Figure 13:**
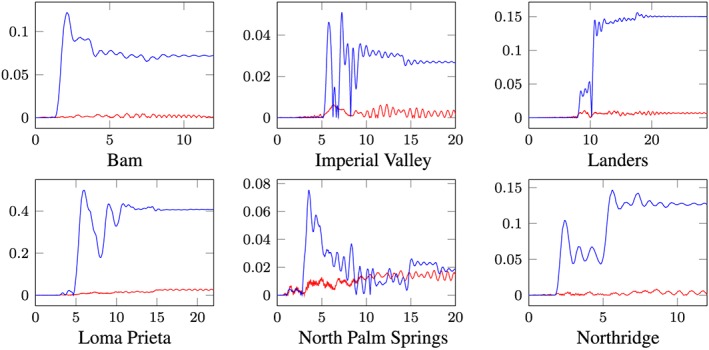
Error evaluation; *x*‐axes ... time [*s*], *y*‐axes ... POD error function *x*_*r*,*POD*_(*t*)[*m*] (red) and modal truncation error function *x*_*r*,*M*_(*t*)[*m*] (blue). [Colour figure can be viewed at wileyonlinelibrary.com]

For the sake of accuracy evaluation a posteriori error analysis is presented. As reference solution to the reduced response functions, that is, solutions by POD reduction and modal truncation, and the solution of the central difference algorithm in the physical coordinate is chosen. The error indicators are

(17a) $$x_{r,POD}(t)=\left|x_{h,CD}(t)-x_{h,POD}(t)\right|,$$

(17b) $$x_{r,M}(t)=\left|x_{h,CD}(t)-x_{h,M}(t)\right|,$$

where *x*_*h*,*CD*_(*t*),*x*_*h*,*POD*_(*t*) and *x*_*h*,*M*_(*t*) are the response functions of the central difference, the POD and the modal truncation responses of the node shown in Figure 1. Figure 13 represents the absolute error of the reduction methods corresponding to analyses provided in Figures 7, 8, 9, 10, 11, 12 as defined in Equation (17).

As depicted in Figure 13, the error by application of the classical modal truncation method is much more considerable than the error produced by the approximation through the POD strategy. Especially, if the structure responds nonlinearly, that is, plastic deformation patterns occur, a huge difference by application of the modal truncation method is observed. Figure 13 provides evidence of the inability of the linear modes to represent elasto‐plastic displacement patterns shown in Figures 4 and 5. Additionally, the applicability and accuracy of the proposed POD strategy seem not to be remarkably sensitive to differences of the peak acceleration response spectra of the excitation time histories in the presented examples. This is seen in the peak acceleration response spectra in the bottom left plot of Figures 7, 8, 9, 10, 11, 12 and the related error functions in Figure 13, where a correlation between those parameters cannot be detected directly. One noticeable point here is that the error functions of the Landers and the Northridge response in Figure 13 seem to be in similar ranges, but the characteristics of the response spectra show considerable differences. Therefore, the proposed strategy demonstrates a high robustness with regard to time history analysis in earthquake engineering and should be of great value in this field.

However, a more extensive evaluation of direct correlations between changes in the spectra of the excitation histories and the quality of the outcome of the POD response is beyond the scope of this paper. Additionally, future research should also include a priori error estimations based on wavelet transformations of the excitation functions. The underlying idea is that earthquake records with similar intensities and frequency contents activate the same nonlinearities and consequently lead to the same POD basis. A publication concerning this strategy appeared quite recently 33.

## 5 Practical Application of the New Approach

**Figure 14:**
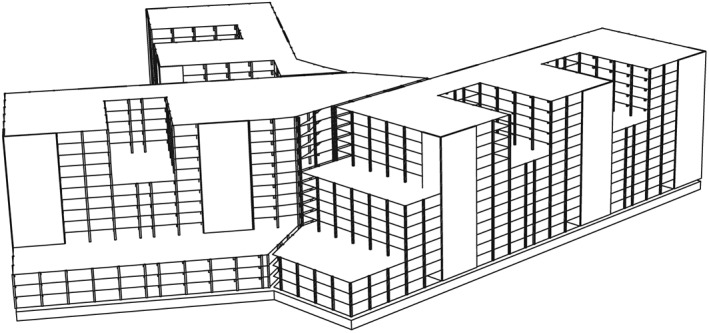
Three‐dimensional visualization of the building construction.

In addition to dealing with the development of the introduced novel POD‐based MOR approach, it is within this section to represent the application of the new proposed MOR strategy on a realistic example. For this purpose, a dynamic structural model of a medical complex, according to its constructional plan, was derived. A schematic three‐dimensional sketch of the building is depicted in Figure 14.

As shown in Figure 14, the building structure exhibits complex geometries. As a result, it seems to make sense to discretize the geometry by a FE model in order to capture the main dynamic specifications.

If such a structure with medical function is located in an earthquake prone region, one way to improve its seismic performance can be realized through base isolation by means of frictional pendulum bearings. Consequently, the analytical simulations demand large computational time and storage because of the presence of nonlinearity imposed by those frictional isolators. In the following, firstly, the structural system specifications and implementation of frictional bearings are presented. Then, the displacement responses to the set of the six earthquake events presented in Table 2 are evaluated. The numerical evaluations compare the new introduced strategy, as an alternative means, with the iterative Newmark integration scheme, which is known as an efficient and exact method.

### 5.1 Structural system and model specifications

**Figure 15:**
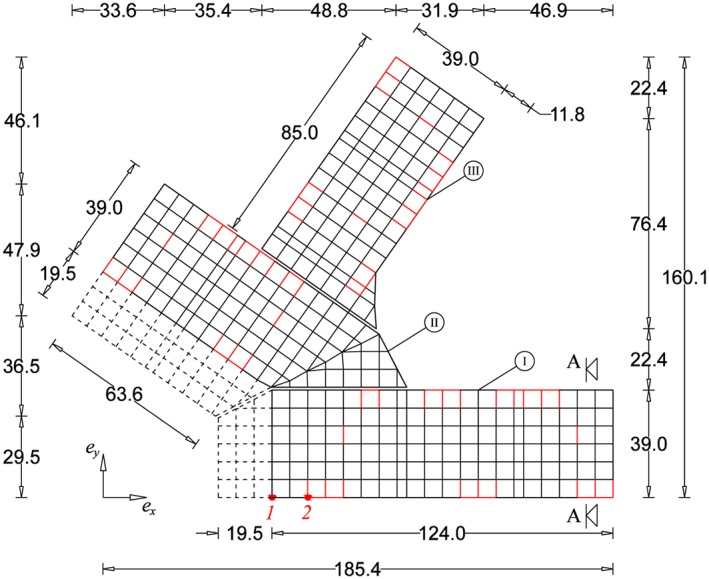
Schematic ground plan, building construction (units in meters), and output nodes 1 and 2 (red marks). [Colour figure can be viewed at wileyonlinelibrary.com]

The building structure consists of three wings, referred to as wing I, II, and III. Figure 15 shows a schematic sketch of the ground plan of the building containing the basic dimensions. The floor slabs of each wing are separate from the others except for the basement slab, which is indiscrete over all three wings. This means that all three wings are coupled through this slab, and they work all together during the earthquake excitations. However, the distance between the wings, which are connected by the basement slab, is about 1.5 m; consequently, contact problems induced by ground motion are not considered in the computations. The grid indicates the location of the columns and the binding beams, and the red lines indicate the location of the shear walls, which are responsible for the lateral reinforcement. The regular distance between the columns is 6.5 m. The building structure has three stories below the ground level, while the highest parts of the building above ground level have 13 stories and the remaining parts have eight stories including the basement levels. Therefore, the plan of the structure is irregular along its height along with the irregularities in the horizontal area. The dashed lines define the area, where the building is only located below the ground. The height of one story is 3m; this leads to a total construction height of 42 m.

**Figure 16:**
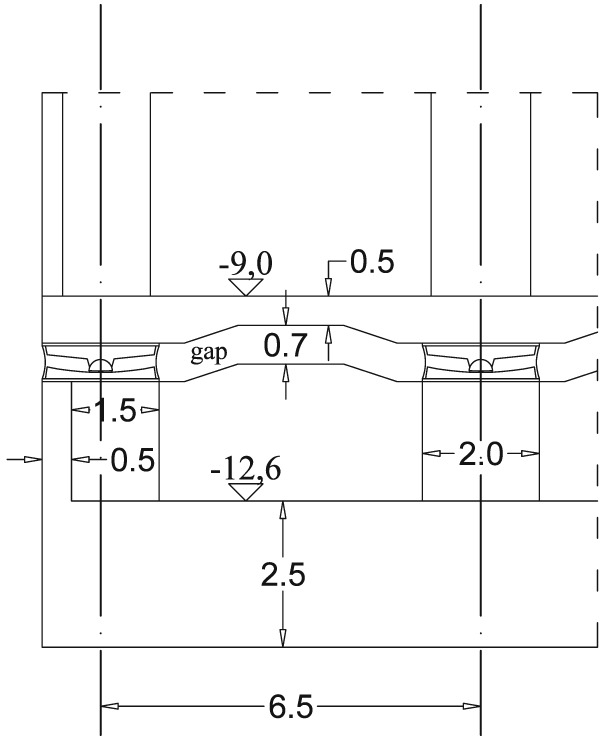
A schematic cutout of the vertical section A‐A of the basement levels presented in Figure 15 (units in meters).

Below the three stories at the basement level, there is the indiscrete slab on the top of the isolators at level of  − 9.00 [m]. Below this slab, along each of the columns, a single friction pendulum (FP) bearing system is attached. Figure 16 depicts a part of the cross section A‐A of the basement level shown in Figure 15. The horizontal diameter of the FP system is 2.00 m. Thus, the dimension of the quadratic cross section of the columns in the basement and FP story is 2.00 × 2.00[*m* ^2^], while in the remaining stories the columns are modeled as quadratic cross sections with the dimensions 0.40 × 0.40[*m* ^2^]. All FP bearings have the same radius of the concave surface, which is equal to 3.00 m.

A representative full‐scale FE model of the building structure was created in the software package slangTNG 34. The shear walls and slabs were modeled by shell/plate elements and the columns and beams by beam elements. A linear elastic material was considered for the modeling of the superstructure (Young's modulus $E=3.5\cdot 10^{10}\;\left[\frac{N}{m^{2}}\right]$, Poisson's ratio *ν* = 0.3[ − ], and density $\rho =2500\;\left[\frac{kg}{m^{3}}\right]$). Nonlinear FP elements, whose implementation in slangTNG is presented in Section 5.2, are assigned below the lowest basement plate of the structure. The total number of DOFs is 33000.

As a result, the superstructure behaves linearly, which is the major reason for implementing base isolator systems for earthquake vibration protection.

### 5.2 Dynamic model of the frictional pendulum element

**Figure 17:**
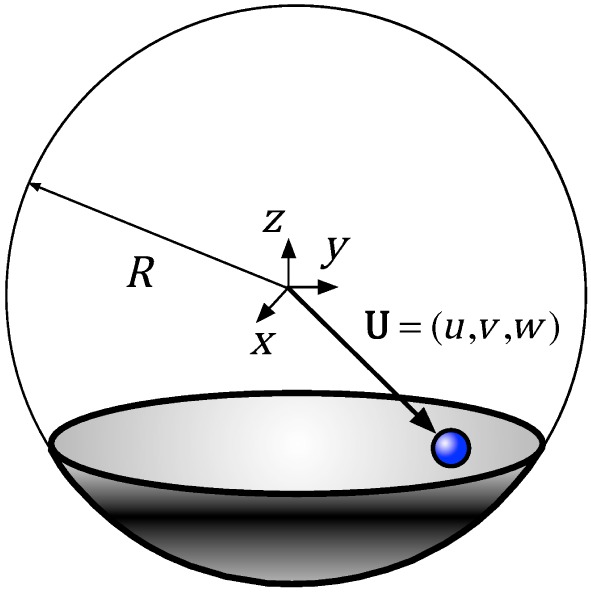
Geometric definition of the friction pendulum element. [Colour figure can be viewed at wileyonlinelibrary.com]

**Figure 18:**
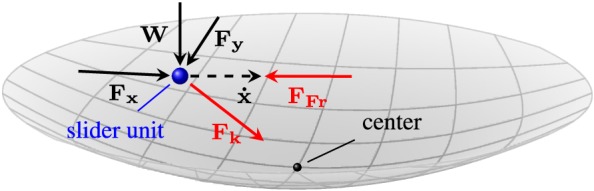
Internal specifications of the friction pendulum element; *F*_*x*_,*F*_*y*_, *N*, recentering force *F*_*k*_, frictional force *F*_*Fr*_. [Colour figure can be viewed at wileyonlinelibrary.com]

This is to present how the FP element in the finite element model of the structure behaves. The geometrical diagram of the FP element, which is realized as a spherical shell, is defined in Figure 17. As depicted, *R* denotes the radius of the concave spherical surface, and the origin of the local coordinate system is chosen to be in the center of the sphere. The position vector of the slider is described by **U** = [*u*,*v*,*w*]^*T*^. Since the desired behavior of the FP element is an in‐plane elasto‐plastic bidirectional action, the change of the vertical position *w* can be neglected. Accordingly, the displacement of the FP element is reduced to an in‐plane motion defined only by the components *u* and *v*, that is, **U** = [*u*,*v*]^*T*^. This simplification makes sense as the radius *R* is much larger relative to the horizontal displacement $|U|\approx \sqrt{u^{2}+v^{2}}$. The equivalent representation of such an element together with the acting forces on it is represented in Figure 18.

The horizontal force equilibrium of the dynamical system is

(18) $$F_{Fr}+F_{k}=F_{ex},\;$$

where *F*_*Fr*_ and *F*_*k*_ are the elasto‐plastic frictional and centring force, and *F*_*ex*_ = [*F*_*x*_,*F*_*y*_]^*T*^ accounts for the interacting horizontal force, which couples the FP element to the super structure.

Following, the force equilibrium is split into two parts as two dynamic situations can occur: situation stick and situation slide. The force equilibrium during the situation stick yields to

(19) $$F_{ex}=\underset{F_{k}}{\underbrace{k_{1}\left\{\begin{array}{l} u\\ v \end{array}\right\}}}+\underset{F_{Fr}}{\underbrace{k_{2}\left\{\begin{array}{l} \Delta u\\ \Delta v \end{array}\right\}}}\;\text{if}\;|F_{ex}-F_{k}|<\mu N.\;$$

This relation renders a linearly‐elastic system, where the friction coefficient *μ* must be a value between 0 and 1. For the computations, this value was taken to be once 4%, and for the second demonstration run of the method it was set equal to 8%. The normal contact force *N* acts orthogonal to the contact area of the slider and the concave surface. The vector **Δ** **U** = [Δ*u*,Δ*v*]^*T*^ defines the radial distance with respect to the current sticking point of the slider if the sticking condition is true. During the situation slide, the FP element is described by the following horizontal force equilibrium:

(20) $$F_{ex}=\underset{F_{k}}{\underbrace{k_{1}\left\{\begin{array}{l} u\\ v \end{array}\right\}}}+\underset{F_{Fr}}{\underbrace{\frac{\mu N}{\left|\dot{U}\right|}\left\{\begin{array}{l} \dot{u}\\ \dot{v} \end{array}\right\}}}\;\text{if}\;|F_{ex}-F_{k}|\geq \mu N,\;$$

where $\dot{U}={\left[\dot{u},\dot{v}\right]}^{T}$ is the velocity vector. In both relations, that is, Equations ((19)) and ((20)), the centring force, $|F_{k}|=k_{1}r=k_{1}\sqrt{u^{2}+v^{2}}$, acts linearly orthogonal to the vertical axis through the deepest point of the surface and the center of the sphere. The fact that the centring force is linear indicates that the spherical shell of the real system is approximated by the paraboloid, whose potential energy increases with $\frac{W}{R}r$ in radial distance from the deepest point, that is, the stiffness is inversely proportional to the radius of the sphere $k_{1}=\frac{W}{R}$.

The frictional force *F*_*Fr*_ is modeled either linearly elastic or elastic‐perfectly plastic as presented in Equations ((19)) and ((20)), respectively. Note that the force corresponding to **Δ** **U** accounts for the elastic behavior of the bearing coating material, in a small elastic range (situation stick, Equation ((19))) and acts towards the current location of the slider (not the center of the concave sphere). Generally, the implementation of a realistic model requires *k*_2_ to be much larger than *k*_1_, that is, *k*_2_ ≫ *k*_1_. During the situation slide, the frictional force acts in opposite direction to the velocity with the magnitude (perfectly plastic) *μ* *N*. This is discussed in Equation ((20)). Another point regarding Equation ((19)) is that the reacting force *N* is assumed to be constant throughout the calculation procedure. This is justified by the following reasons: firstly, just *x* and *y* components of the exciting ground motion are taken into account for the computations. Secondly, the motion has already been simplified to be planar and therefore no additional force component due to vertical motion is generated. Finally, in our preliminary analysis, the uplift force on the isolator slap was observed to be extremely small in comparison with the downward force because of the weight of the structure. Considering the above‐mentioned fact together with the force diagram given in in Figure 18, follows that the normal contact force *N* is approximately constant and equal to the weight induced force of the super structure, *W*, that is, *N* = *W* in Equation ((20)).

The FP bearing element governed by Equations ((18))–((20)) has been implemented in the software package slangTNG 34. For a comparable study on this implemented friction pendulum system, the experimental work of Mosqueda *et al.* 35 is suggested. Concerning the computations in this paper, the friction coefficient *μ* of the FP slider is assumed to be a constant value, as discussed previously. However, *μ* is indeed a value dependent on velocity, pressure, and temperature. Recent publications dealing with this issue are authored by Castaldo and Tubaldi 36, as well as Kumar *et al.* 37. Regarding the proposed new strategy, it is expected that the implementation of a friction coefficient, which depends on velocity, pressure, and temperature, would lead to comparable results concerning accuracy and speed of the reduced order model. In other words, it is envisiged that a specific nonlinear model of the FP bearing element with variable friction coefficient does not influence the overall behavior of the reduced order model in comparison with the full model, that is, the effectiveness of the model order reduction procedure. In every time step, the vector of the inner restoring forces in Equation ((16)) has to be evaluated in the full (physical) coordinate anyway.

For additional information about friction pendulum systems, the reader is referred to the literature (e.g., 38, 39, 40, 41, 42, 43). More detailed examination of this topic would lead beyond the scope of this paper, which should focus more on the methodical extension of the new MOR strategy as well as the application on a complex realistic system.

### 5.3 Numerical evaluation

The evaluation of the MOR strategy applied to the realistic building structure is dealt with displacement response calculations to the six different earthquake excitations presented in Table 1. The calculation outputs are presented by the in‐plane motion response of the slider at the red marked node (node 1: coordinates [19.5,0.0, − 9.5]^*T*^[*m*]) in Figure 15 in *x* and *y* direction. As this node defines the location of a moving friction pendulum, it shows directly the nonlinear response behavior of the system. Additionally, a second output node (node 2: coordinates [32.5,0.0,*h*]^*T*^[*m*]) is chosen. Here, the coordinate *h* stands for every possible story of the building structure. According to *h* = 32.5[*m*], the maximum acceleration of the roof is presented and according to each story (h=−9.5,−6.5,−3.5,…,32.5[m]), the maximum drift responses are shown.

The numerical demonstrations are performed by assuming two different friction coefficients for the FP‐isolators, namely 4% and 8%. The methodology applied to the realistic example is equivalent to the academic example, which is presented in Section 4. Therefore, the main focus in the next sections is on the presentation of the numerical results.

**Figure 19:**
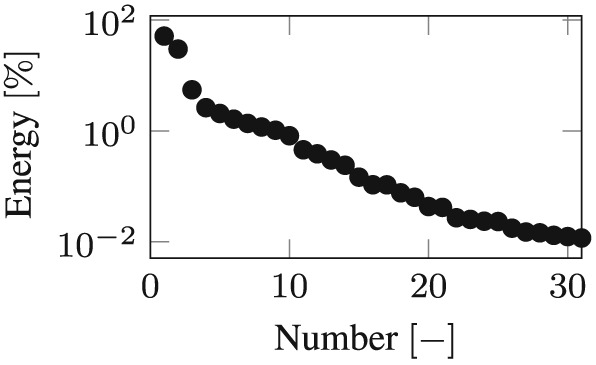
Proper orthogonal decomposition values; friction coefficient *μ* = 0.04[ − ] (logarithmic scale).

**Figure 20:**
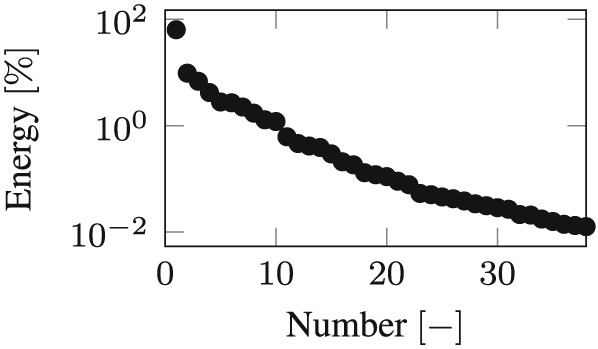
Proper orthogonal decomposition values; friction coefficient *μ* = 0.08[ − ] (logarithmic scale).

Integration over the whole Bam earthquake by the Newmark method in the physical coordinate leads to the snapshot response function *x*_*S*_(*t*) and consequently to the snapshot matrix *X*_*S*_, which contains 400 snapshots in equidistant time intervals. Following, the evaluation of the left singular vectors of the snapshot matrix leads to the POD modes and its singular values to the POD values in descending order. The number of POD modes that have to be taken into account in order to capture 99.99% of the total energy was evaluated to be 31 for the calculations concerning the friction coefficient of 4% and 38 concerning the friction coefficient of 8%. The logarithmic plots of the singular values (POD values) for both friction coefficients dependent on the corresponding energy are shown in the Figures 19 and 20.

**Figure 21:**
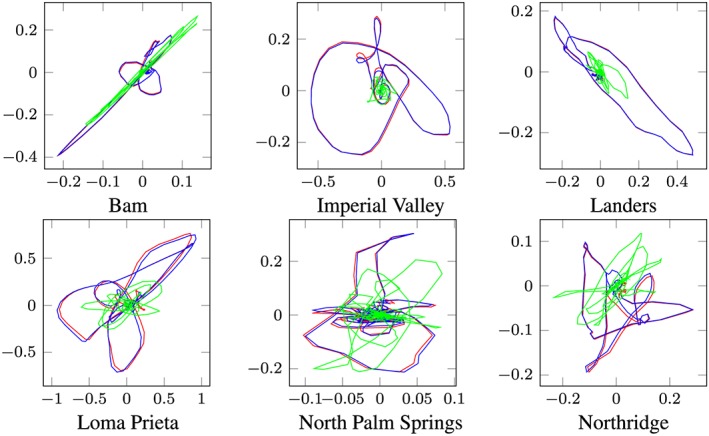
Planar position of the friction pendulum‐element (output node 1) over the time period of excitation; friction coefficent *μ* = 0.04; *x*‐axes ... slider displacement in *x*‐direction [*m*], *y*‐axes ... slider displacement *y*‐direction [*m*]; blue line ... full response; red line ... proper orthogonal decomposition response; green line ... modal truncation response. [Colour figure can be viewed at wileyonlinelibrary.com]

Time integration in the 31 and 38‐dimensional POD reduced subspace over the whole Bam earthquake produces the POD reduced response. After back transformation into the physical coordinate, the reduced response must be compared with the benchmark solution (full Newmark response). The response motion of the slider (output node 1) of the full and the POD‐reduced calculation is depicted in Figures 21(a) (friction coefficient of 4%) and 22(a) (friction coefficient of 8%). As clearly shown, a reliable approximation of the benchmark solution is achieved; therefore, nonlinear effects can be represented. In the next step, the responses to the remaining earthquakes are evaluated in the reduced subspaces, as well as the full reference solutions by application of the standard Newmark algorithm.

**Figure 22:**
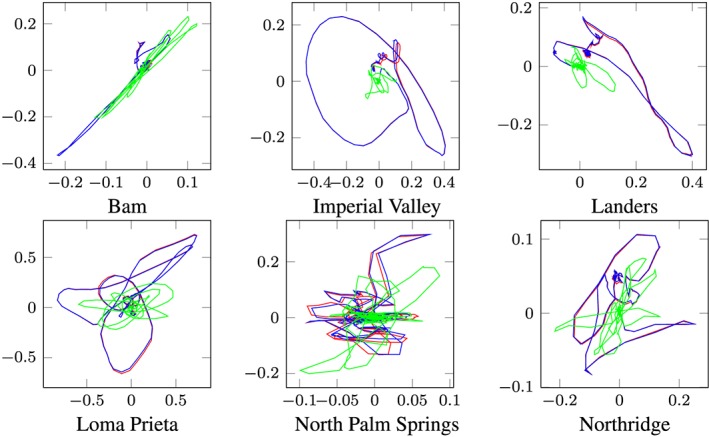
Planar position of the friction pendulum‐element (output node 1) over the time period of excitation; friction coefficent *μ* = 0.08; *x*‐axes ... slider displacement in *x*‐direction [*m*], *y*‐axes ... slider displacement *y*‐direction [*m*]; blue line ... full response; red line ... proper orthogonal decomposition response; green line ... modal truncation response. [Colour figure can be viewed at wileyonlinelibrary.com]

The first 30 modes are computed according to a linearization of the slider element. The boundary conditions below each slider element are fixed. The required linearization of the slider element is realized by taking the initial stiffness matrix of the system into account, similarly to the linearization assumption of the academic example. This inevitably leads to an over‐estimation of the slider stiffness, although the vector of the inner restoring forces is calculated.

The red lines in Figures 21 and 22 show the POD reduced responses and the blue lines show the full reference solutions obtained by the standard Newmark method. As depicted in these figures, accurate approximations are achieved for both assumptions of the friction coefficients (note: the lines cover each other). Additionally, it is clearly shown in these figures that the modal truncation method (green lines) fails to approximate the full solution.

**Figure 23:**
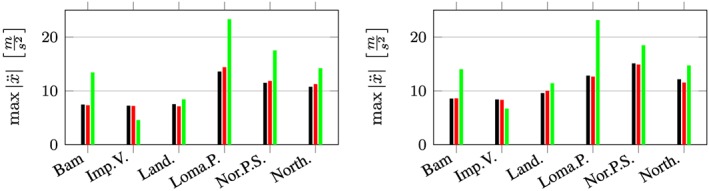
Maximum acceleration at the roof (above output node 2); left subplot: *μ* = 0.04; right subplot: *μ* = 0.08; full Newmark (black), proper orthogonal decomposition response (red), and modal truncation (green). [Colour figure can be viewed at wileyonlinelibrary.com]

**Figure 24:**
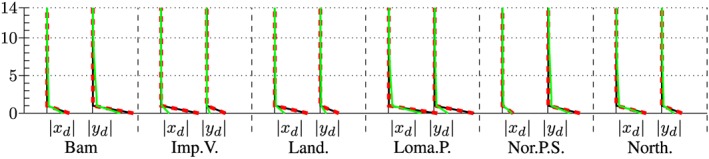
Maximum drift of the stories above the output node 2 in *x* and *y* direction; *μ* = 0.04; including the slider drift in the ground floor 0, full Newmark (black), proper orthogonal decomposition (red dashed), and modal truncation (green). [Colour figure can be viewed at wileyonlinelibrary.com]

**Figure 25:**
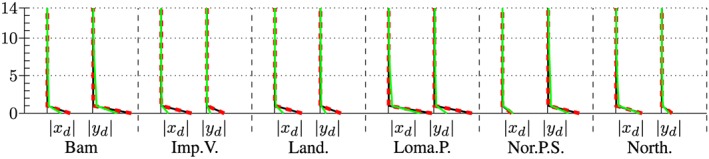
Maximum drift of the stories above the output node 2 in *x* and *y* direction; *μ* = 0.08; including the slider drift in the ground floor 0, full Newmark (black), proper orthogonal decomposition (red dashed), and modal truncation (green). [Colour figure can be viewed at wileyonlinelibrary.com]

**Figure 26:**
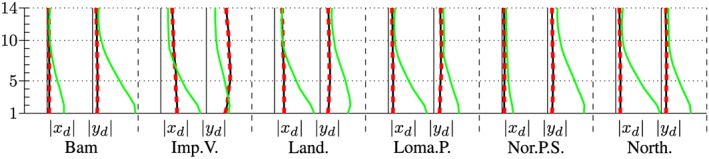
Maximum drift of the stories above the output node 2 in *x* and *y* direction; *μ* = 0.04; excluding the slider drift in the ground floor 0, full Newmark (black), proper orthogonal decomposition (red dashed), and modal truncation (green). [Colour figure can be viewed at wileyonlinelibrary.com]

**Figure 27:**
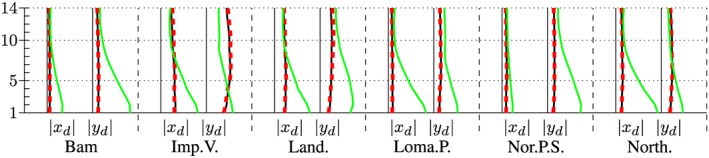
Maximum drift of the stories above the output node 2 in *x* and *y* direction; *μ* = 0.08; excluding the slider drift in the ground floor 0, full Newmark (black), proper orthogonal decomposition (red dashed), and modal truncation (green). [Colour figure can be viewed at wileyonlinelibrary.com]

The maximum accelerations at the roof in output node 1 are presented in the left and right subplot of Figure 23. Additionally, the maximum drifts between all floors above output node 2 are presented in Figures 24 and 25. As for both friction coefficients, the slider drift itself (drift in the floor 0) is generally much larger (which is actually the purpose of base isolation). Therefore, for each of the friction coefficients, a second figure is presented in order to create the possibility to visualize the results, not only in the slider but also in the hospital building itself. This is realized in Figures 26 and 27. The numerical values of the drifts of the stories 0 (slider drift), 1, 5, 10, and 14 are presented in Tables 2 (for friction coefficient of 0.04) and 3 (for friction coefficient of 0.08).

**Table 2:** *x*‐component of the maximum drift above output node 2 in meters; *μ* = 0.04; corresponding to the Newmark response function.

Story	Bam *x*_*d*_	Imperial Valley *x*_*d*_	Landers *x*_*d*_	Loma Prieta *x*_*d*_	North Palm Springs *x*_*d*_	North. Rinaldi *x*_*d*_
0	0.212	0.556	0.482	0.899	0.107	0.287
1	0.00201	0.00618	0.00449	0.00874	0.00215	0.00293
5	0.00223	0.00587	0.00452	0.00947	0.00239	0.00304
10	0.00182	0.00488	0.00385	0.00836	0.00225	0.00259
14	0.00167	0.00439	0.00346	0.00745	0.00190	0.00234

**Table 3:** *x*‐component of the maximum drift above output node 2 in meters; *μ* = 0.08; corresponding to the Newmark response function.

Story	Bam *x*_*d*_	Imperial Valley *x*_*d*_	Landers *x*_*d*_	Loma Prieta *x*_*d*_	North Palm Springs *x*_*d*_	North Rinaldi *x*_*d*_
0	0.219	0.451	0.399	0.832	0.0933	0.250
1	0.00247	0.00562	0.00514	0.00810	0.00398	0.00370
5	0.00256	0.00529	0.00546	0.00836	0.00463	0.00444
10	0.00243	0.00448	0.00477	0.00732	0.00418	0.00400
14	0.00209	0.00391	0.00426	0.00656	0.00368	0.00352

It is expected that the proposed new strategy is also applicable to different types of nonlinearities, for example, large deformations, viscoelasticity, viscoplasticity, hyperelasticity, and so on. In addition, concerning seismic protection, it is expected that the proposed new strategy is also applicable to smart structures with shape memory alloy‐based seismic damping and isolation tools. The new strategy should produce response approximations with comparable accuracy to the nonlinearites already presented in this paper. However, it is also of utmost importance here to emphasize the fact that the nonlinear deformation patterns, namely the special hysteretic behavior dependent on superelasticity, temperature, and memory effect, must be captured in the snapshot matrix.

## 6 Conclusion

In this paper, a MOR strategy, which is applicable to the dynamic response analysis of linear and nonlinear structural systems is presented. Usually, the analysis of building structures with complex geometries makes the engineer to create a FE model with a large number of DOFs, which is associated with computational effort in the response analysis. Therefore, the goal of this paper is to provide a new MOR strategy that is simple in application, but also very effective even in the presence of nonlinearities for problems in the field of earthquake engineering and structural dynamics. This strategy is extended based on the POD method to derive a proper transformation matrix in order to transform the nonlinear systems into another low‐dimensional subspace, which demands considerably less computational effort for the response calculation. Once the transformation matrix is derived, the approach of the strategy is similar to the method of modal truncation for linear systems. The presentation of the novel approach comes along with a simple and illustrative example that points out the benefit compared with standard methods as modal truncation.

In addition to the development of the MOR strategy, its application for the response calculation of a realistic numerical nonlinear example is demonstrated. The example is the displacement response calculation of a building structure serving as a medical complex, which is base‐isolated by FP bearing systems excited by six earthquake excitations. In order to evaluate the accuracy of the introduced approach, the exact structural responses are also calculated. Numerical evaluations show that reliable approximations can be achieved if nonlinear response patterns of the structure are already captured in the POD snapshots to extract the transformation matrix. The advantage of this strategy is obvious that the transformation matrix is derived just once, and it can be used for response calculation of the structure under different earthquake excitations.

Another substantial advantage of the introduced MOR concerns the speed of the response calculations. Firstly, compared with the basic central difference algorithm, the new introduced strategy has a much larger critical time step. Secondly, compared with the Newmark method, which allows usually larger time steps, no iteration procedure is required.

## Acknowledgements

The authors would like to acknowledge the financial support from the Austrian Science Funds (FWF) as part of the Vienna Doctoral Programme on Water Resource Systems (DK‐plus W1219‐N22). This research has also been supported by Stiftung Maurer Söhne (Forschungsförderung Technische Dynamik), which is gratefully acknowledged by the authors. Special thanks from the authors is dedicated to Prof. Bernd Markert, head of the Institute of General Mechanics at the RWTH Aachen University, for his critical feedback and constant support.

## References

[1] Rega G . Dimension reduction of dynamical systems: methods, models, applications. Nonlinear Dynamics 2005; 41:1–15.

[2] Koutsovasilis P , Beitelschmidt M . Comparison of model reduction techniques for large mechanical sytems. Multibody System Dynamics 2008; 20:111–128.

[3] Kosambi D . Statistics in function space. Journal of the Indian Mathematical Society 1943; 7:76–88.

[4] Karhunen K . Über lineare methoden in der wahrscheinlichkeitsrechnung. *PhD thesis*, University of Helsinki, 1947.

[5] Loeve M . Fonctions al eatoire de second ordre. Revue Scientifique 1946; 48:195–206.

[6] Cusumano JP , Sharkady MT , Kimble BW . Spatial coherence measurements of a chaotic flexible‐beam impact oscillator. American Society of Mechanical Engineers, Aerospace Division (Publication) AD 1993; 33:13–22.

[7] Feeny BF , Kappagantu R . On the physical interpretation of proper orthogonal modes in vibrations. Journal of Sound and Vibration 1998; 211:607–616.

[8] Kappagantu R , Feeny BF . An “optimal” modal reduction of a system with frictional excitation. Journal of Sound and Vibration 1999; 224:863–877.

[9] Kappagantu R , Feeny BF . Part 1: dynamical characterization of a frictionally excited beam. Nonlinear Dynamics 2000; 22:317–333.

[10] Kappagantu R , Feeny BF . Part 2: proper orthogonal modal modeling of a frictionally excited beam. Nonlinear Dynamics 2000; 23:1–11.

[11] Liang YC , Lee HP , Lim SP , Lin WZ , Lee KH , Wu CG . Proper orthogonal decomposition and its applications–Part 1: theory. Proper Orthogonal Decomposition Theory. Journal of Sound and Vibration 2002; 252:527–544.

[12] Kerschen G , Golivani JC . Physical interpretation of the proper orthogonal modes using the singular value decomposition. Journal of Sound and Vibration 2002; 249:849–865.

[13] Kerschen G , Golivani JC . A model updating strategy of non‐linear vibrating structures. International Journal of Numerical Methods in Engineering 2003; 60:2147–2164.

[14] Qu Z , Shi Y , Hua H . A reduced‐order modeling technique for tall buildings with active tuned mass damper. Earthquake Engineering and Structural Dynamics 2001; 30:349–362.

[15] Schemann AG , Smith HA . Vibration control of cable‐stayed bridges ‐ part 1: modelling issues. Earthquake Engineering and Structural Dynamics 1998; 27:811–824.

[16] Krysl P , Lall S , Marsden JE . Dimensional model reduction in non‐linear finite element dynamics of solids and structures. International Journal for Numerical Methods in Engineering IJNME 2001; 51:479–504.

[17] Tubino F , Carassale L , Solari G . Seismic response of multi‐supported structures by proper orthogonal decomposition. Earthquake Engineering and Structural Dynamics 2003; 32:1639–1654.

[18] Bucher C . Stabilization of explicit time integration by modal reduction. In *Proceedings, Trends in Computational Mechanics,* Barcelona: CINME, 2001.

[19] Gutierrez E , Cela JJL . Improving explicit time integration by modal truncation techniques. Earthquake Engineering And Structural Dynamics 1998; 27:1541–1557.

[20] Gutierrez E , Zaldivar JM . The application of Karhunen‐Loeve, or principal component analysis method, to study the non‐linear seismic response of structures. Earthquake Engineering and Structural Dynamics 2000; 29:1261–1286.

[21] Bamer F , Bucher C . Application of the proper orthogonal decomposition for linear and nonlinear structures under transient excitation. Acta Mechanica 2012; 223:2549–2563.

[22] Pacific Earthquake Engineering Research Center (PEER). Homepage: http://nisee.berkeley.edu.

[23] Chopra AK . Dynamics of Structures, Theory and Applications to Earthquake Engineering. Prentice Hall: New Jersey, 2001.

[24] Koutsovasilis P . Comparison of model reduction techniques for large mechanical sytems. Multibody System Dynamics 2008; 20:111–128.

[25] Ibrahimbegovic A , Wilson EL . A methodology for dynamic analysis of linear structure‐foundation systems with local nonlinearities. International Journal for Earthquake Engineering and Structural Dynamics 1990; 19:1197–1208.

[26] Ibrahimbegovic A , Wilson EL . Efficient computational procedures for systems with local nonlinearities. Engineering Computations 1992; 9:385–398.

[27] Chatterjee A . An introduction to the proper orthogonal decomposition. Current Science 2000; 78:808–817.

[28] Holmes P , Lumley JL , Berkooz G . Turbulence, Coherent Structures, Dynamical Systems and Symmetry. Cambridge University Press: Cambridge, 1996.

[29] Qu ZQ . Model Order Reduction Techniques with Application in Finite Element Analysis. Springer: London, 2004. Limited.

[30] Han S , Feeny B . Application of proper orthogonal decomposition to structural vibration analysis. Mechanical Systems and Signal Processing 2003; 17:989–1001.

[31] Kerschen G , Golinval C , Vakakis F , Bergman . The method of proper orthogonal decomposition for dynamical characterization and order reduction of mechanical systems: an overview. Nonlinear Dynamics 2005; 41:147–169.

[32] Giaralis A , Spanos PD . Wavelet‐based response spectrum compatible synthesis of accelerograms‐eurocode application (EC8). Soil Dynamics and Earthquake Engineering 2009; 29:219–235.

[33] Podrouzek J , Bucher C , Deodatis G . Identification of critical samples of stochastic processes towards feasible structural reliability applications. Structural Safety 2014; 47:39–47.

[34] Christian Bucher . Open source structural dynamics and FE software (slangTNG). Homepage: http://info.tuwien.ac.at/bucher/Private/Welcome.html.

[35] Mosqueda G , Whittaker A , Fenves G . Characterization and modeling of friction pendulum bearings subjected to multiple components of excitation. Journal of Structural Engineering ASCE 2004; 130:433–442.

[36] Castaldo P , Tubaldi E . Influence of FPS bearing properties on the seismic performance of base‐isolated structures. Earthquake Engineering and Structural Dynamics 2015; 44:2817–2836.

[37] Kumar M , Whittaker AS , Constantinou C . Characterizing friction in sliding isolation bearings. Earthquake Engineering and Structural Dynamics 2015; 44:1409–1425.

[38] Almazan J , Llera J , Inaudi J . Modelling aspects of structures isolated with the frictional pendulum system. Earthquake Engineering and Structural Dynamics 1998; 27:845–867.

[39] Llera J , Almazan J . An experimental study of nominally symmetric and asymmetric structures isolated with the FPS. Earthquake Engineering and Structural Dynamics 2003; 32:891–918.

[40] Ordonez D , Foti D , Bozzo L . Comparative study of the inelastic response of base isolated buildings. Earthquake Engineering and Structural Dynamics 2003; 32:151–164.

[41] Ryan K , Chopra AK . Estimating the seismic displacement of friction pendulum isolators based on non‐linear response history analysis. Earthquake Engineering and Structural Dynamics 2004; 33:359–373.

[42] Ray T , Sarlis A , Reinhorn A , Constantinou M . Hysteretic models for sliding bearings with varying frictional force. Earthquake Engineering and Structural Dynamics 2013; 42:2341–2360.

[43] Bucher C . Probability‐based optimal design of friction‐based seismic isolation devices. Structural Safety 2009; 31:500–507.

